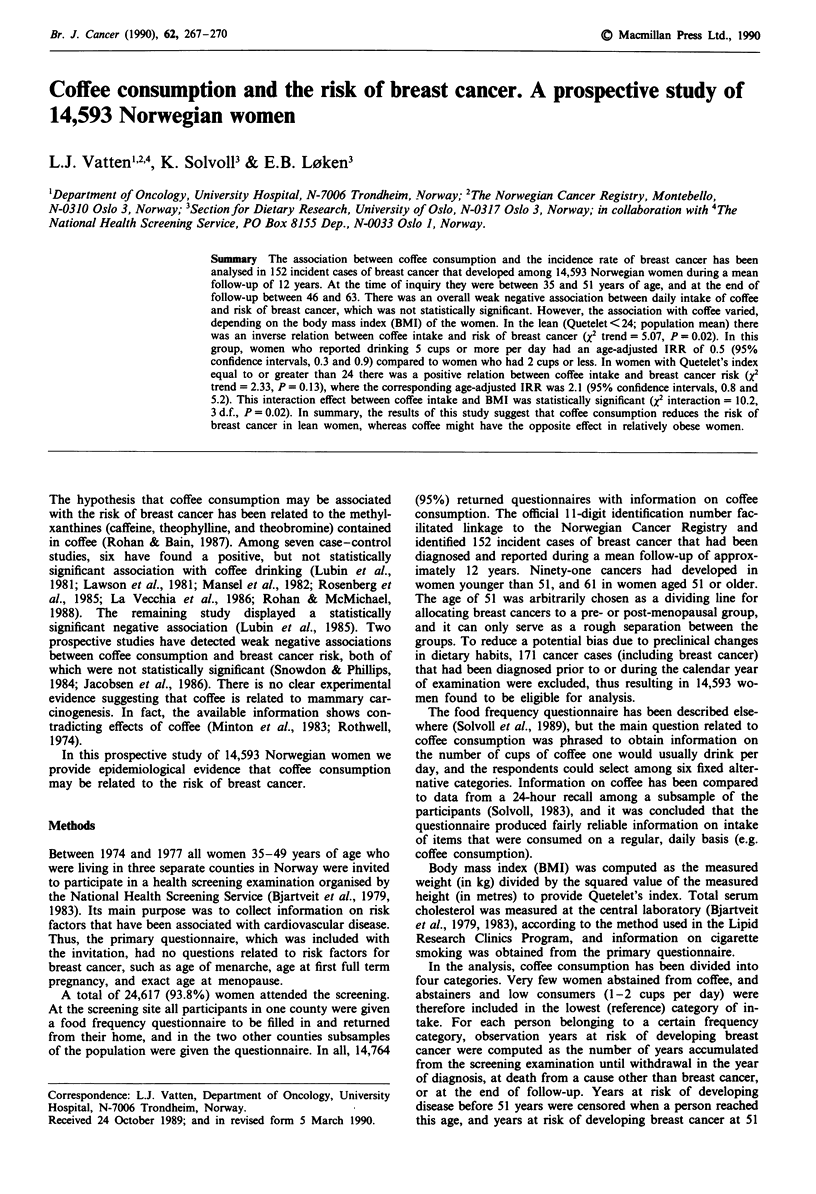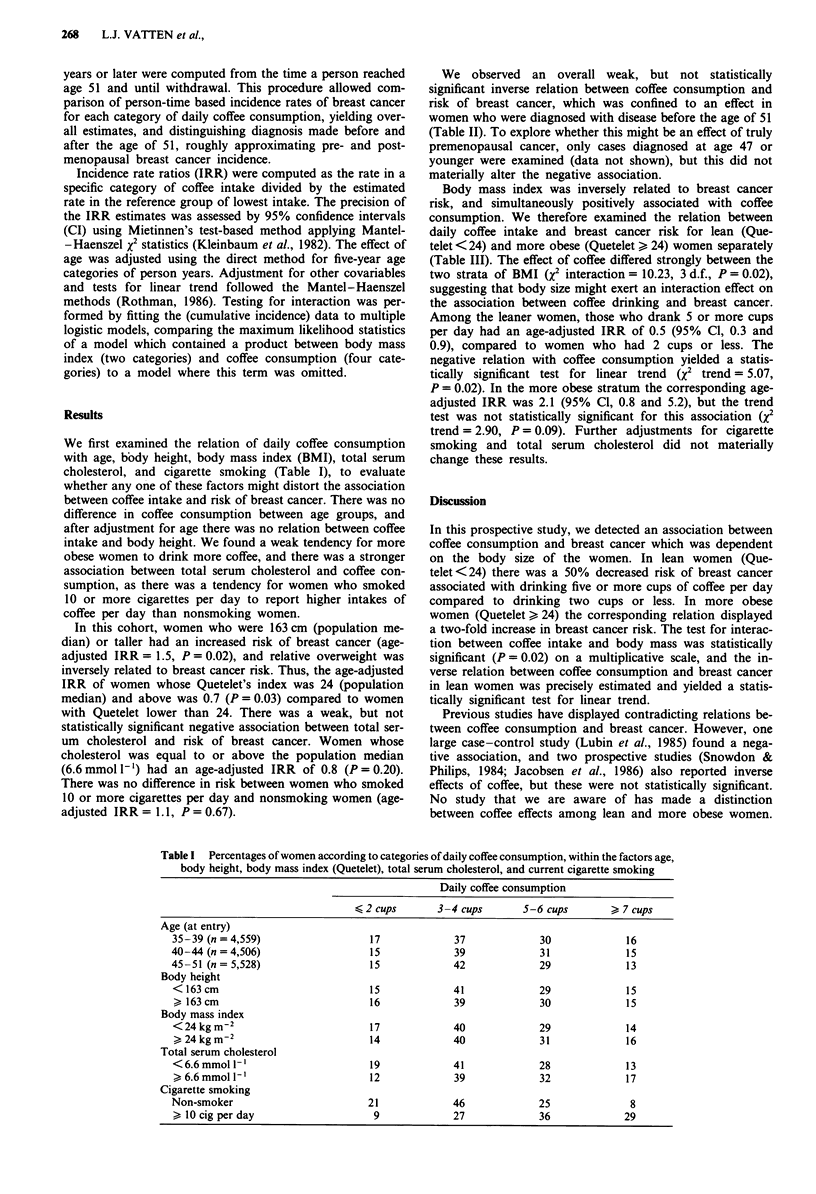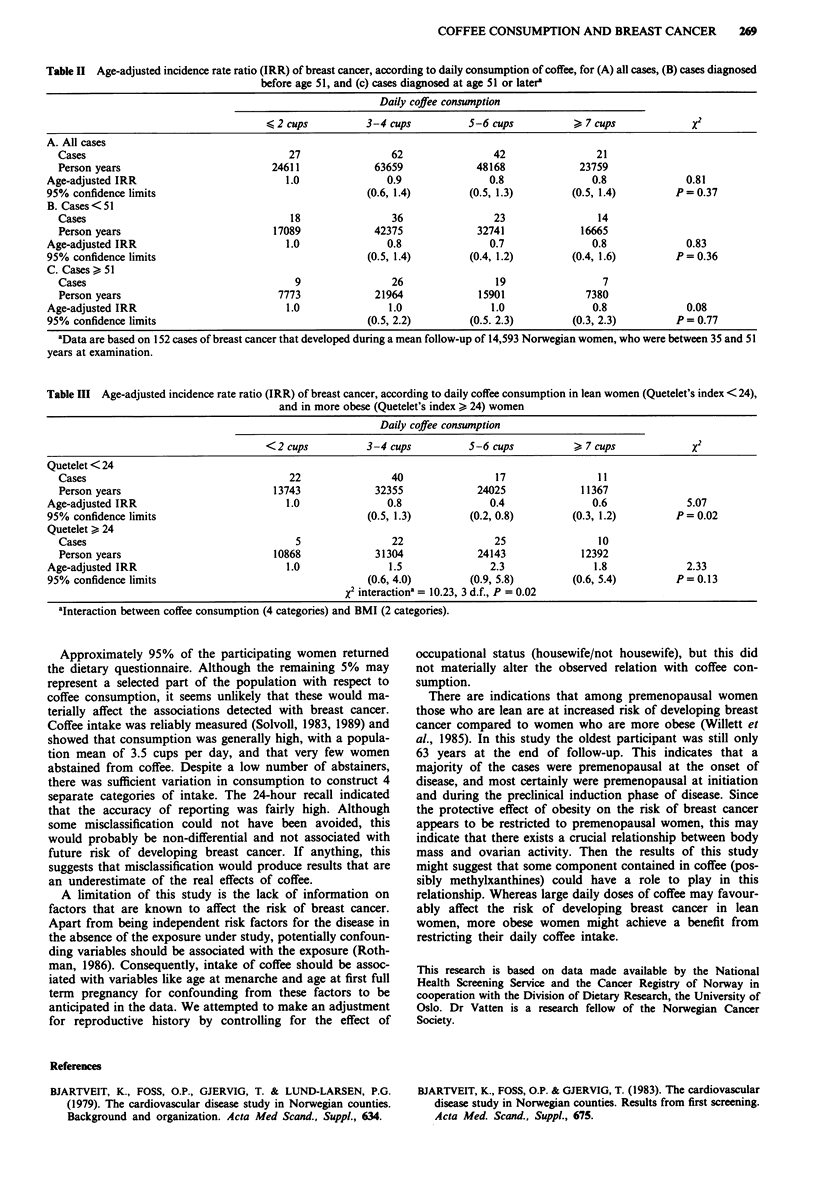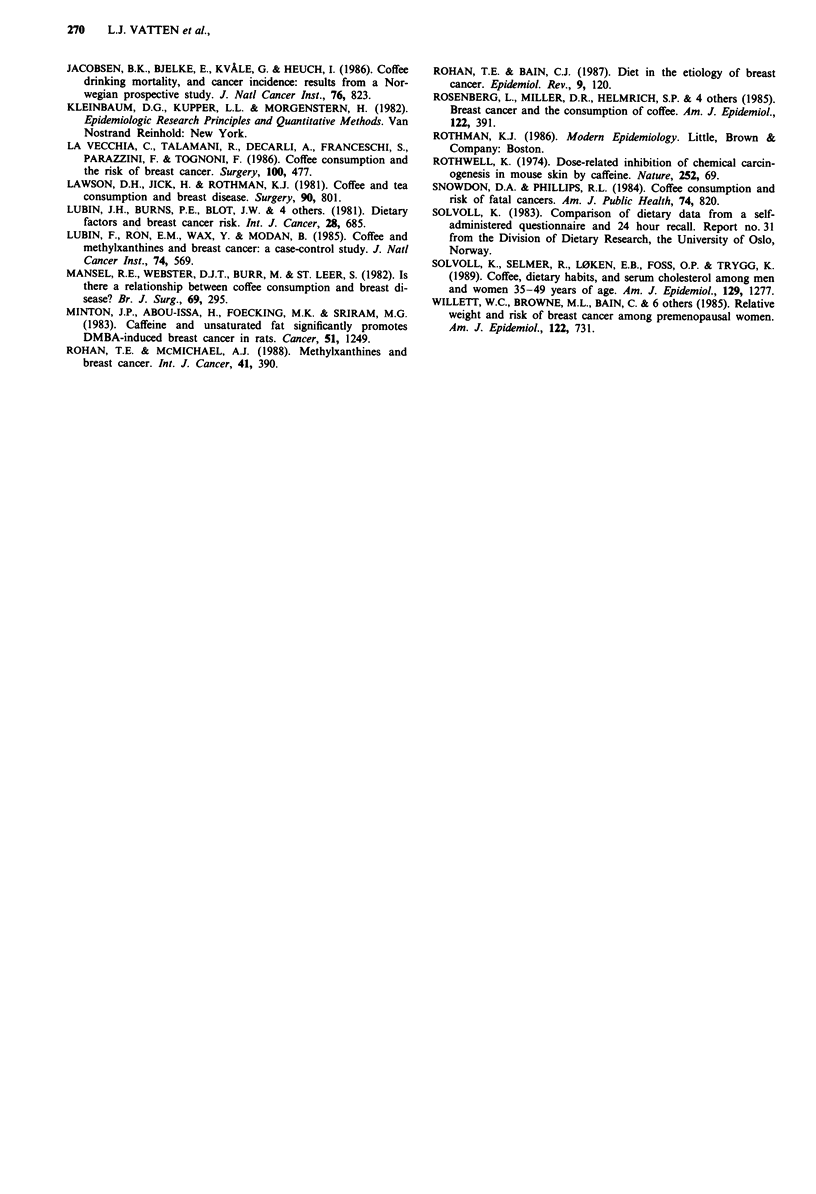# Coffee consumption and the risk of breast cancer. A prospective study of 14,593 Norwegian women.

**DOI:** 10.1038/bjc.1990.274

**Published:** 1990-08

**Authors:** L. J. Vatten, K. Solvoll, E. B. Løken

**Affiliations:** Department of Oncology, University Hospital, Trondheim, Norway.

## Abstract

The association between coffee consumption and the incidence rate of breast cancer has been analysed in 152 incident cases of breast cancer that developed among 14,593 Norwegian women during a mean follow-up of 12 years. At the time of inquiry they were between 35 and 51 years of age, and at the end of follow-up between 46 and 63. There was an overall weak negative association between daily intake of coffee and risk of breast cancer, which was not statistically significant. However, the association with coffee varied, depending on the body mass index (BMI) of the women. In the lean (Quetelet less than 24; population mean) there was an inverse relation between coffee intake and risk of breast cancer (chi 2 trend = 5.07, P = 0.02). In this group, women who reported drinking 5 cups or more per day had an age-adjusted IRR of 0.5 (95% confidence intervals, 0.3 and 0.9) compared to women who had 2 cups or less. In women with Quetelet's index equal to or greater than 24 there was a positive relation between coffee intake and breast cancer risk (chi 2 trend = 2.33, P = 0.13), where the corresponding age-adjusted IRR was 2.1 (95% confidence intervals, 0.8 and 5.2). This interaction effect between coffee intake and BMI was statistically significant (chi 2 interaction = 10.2, 3 d.f., P = 0.02). In summary, the results of this study suggest that coffee consumption reduces the risk of breast cancer in lean women, whereas coffee might have the opposite effect in relatively obese women.


Br. J. Cancer (1990), 62, 267-270 © Macmillan Press Ltd., 1990

### Coffee consumption and the risk of breast cancer. A prospective study of 14,593 Norwegian women

L.J. Vatten[1,2,4], K. Solvoll[3] & E.B. Løken[3]

[1]*Department of Oncology, University Hospital, N-7006 Trondheim, Norway;* [2]*The Norwegian Cancer Registry, Montebello, N-0310 Oslo 3, Norway;* [3]*Section for Dietary Research, University of Oslo, N-0317 Oslo 3, Norway; in collaboration with* [4]*The National Health Screening Service, PO Box 8155 Dep., N-0033 Oslo 1, Norway.*

**Summary** The association between coffee consumption and the incidence rate of breast cancer has been analysed in 152 incident cases of breast cancer that developed among 14,593 Norwegian women during a mean follow-up of 12 years. At the time of inquiry they were between 35 and 51 years of age, and at the end of follow-up between 46 and 63. There was an overall weak negative association between daily intake of coffee and risk of breast cancer, which was not statistically significant. However, the association with coffee varied, depending on the body mass index (BMI) of the women. In the lean (Quetelet < 24; population mean) there was an inverse relation between coffee intake and risk of breast cancer ($\chi^2$ trend = 5.07, $P = 0.02$). In this group, women who reported drinking 5 cups or more per day had an age-adjusted IRR of 0.5 (95% confidence intervals, 0.3 and 0.9) compared to women who had 2 cups or less. In women with Quetelet's index equal to or greater than 24 there was a positive relation between coffee intake and breast cancer risk ($\chi^2$ trend = 2.33, $P = 0.13$), where the corresponding age-adjusted IRR was 2.1 (95% confidence intervals, 0.8 and 5.2). This interaction effect between coffee intake and BMI was statistically significant ($\chi^2$ interaction = 10.2, 3 d.f., $P = 0.02$). In summary, the results of this study suggest that coffee consumption reduces the risk of breast cancer in lean women, whereas coffee might have the opposite effect in relatively obese women.

The hypothesis that coffee consumption may be associated with the risk of breast cancer has been related to the methylxanthines (caffeine, theophylline, and theobromine) contained in coffee (Rohan & Bain, 1987). Among seven case-control studies, six have found a positive, but not statistically significant association with coffee drinking (Lubin *et al.*, 1981; Lawson *et al.*, 1981; Mansel *et al.*, 1982; Rosenberg *et al.*, 1985; La Vecchia *et al.*, 1986; Rohan & McMichael, 1988). The remaining study displayed a statistically significant negative association (Lubin *et al.*, 1985). Two prospective studies have detected weak negative associations between coffee consumption and breast cancer risk, both of which were not statistically significant (Snowdon & Phillips, 1984; Jacobsen *et al.*, 1986). There is no clear experimental evidence suggesting that coffee is related to mammary carcinogenesis. In fact, the available information shows contradicting effects of coffee (Minton *et al.*, 1983; Rothwell, 1974).

In this prospective study of 14,593 Norwegian women we provide epidemiological evidence that coffee consumption may be related to the risk of breast cancer.

### Methods

Between 1974 and 1977 all women 35-49 years of age who were living in three separate counties in Norway were invited to participate in a health screening examination organised by the National Health Screening Service (Bjartveit *et al.*, 1979, 1983). Its main purpose was to collect information on risk factors that have been associated with cardiovascular disease. Thus, the primary questionnaire, which was included with the invitation, had no questions related to risk factors for breast cancer, such as age of menarche, age at first full term pregnancy, and exact age at menopause.

A total of 24,617 (93.8%) women attended the screening. At the screening site all participants in one county were given a food frequency questionnaire to be filled in and returned from their home, and in the two other counties subsamples of the population were given the questionnaire. In all, 14,764 (95%) returned questionnaires with information on coffee consumption. The official 11-digit identification number facilitated linkage to the Norwegian Cancer Registry and identified 152 incident cases of breast cancer that had been diagnosed and reported during a mean follow-up of approximately 12 years. Ninety-one cancers had developed in women younger than 51, and 61 in women aged 51 or older. The age of 51 was arbitrarily chosen as a dividing line for allocating breast cancers to a pre- or post-menopausal group, and it can only serve as a rough separation between the groups. To reduce a potential bias due to preclinical changes in dietary habits, 171 cancer cases (including breast cancer) that had been diagnosed prior to or during the calendar year of examination were excluded, thus resulting in 14,593 women found to be eligible for analysis.

The food frequency questionnaire has been described elsewhere (Solvoll *et al.*, 1989), but the main question related to coffee consumption was phrased to obtain information on the number of cups of coffee one would usually drink per day, and the respondents could select among six fixed alternative categories. Information on coffee has been compared to data from a 24-hour recall among a subsample of the participants (Solvoll, 1983), and it was concluded that the questionnaire produced fairly reliable information on intake of items that were consumed on a regular, daily basis (e.g. coffee consumption).

Body mass index (BMI) was computed as the measured weight (in kg) divided by the squared value of the measured height (in metres) to provide Quetelet's index. Total serum cholesterol was measured at the central laboratory (Bjartveit *et al.*, 1979, 1983), according to the method used in the Lipid Research Clinics Program, and information on cigarette smoking was obtained from the primary questionnaire.

In the analysis, coffee consumption has been divided into four categories. Very few women abstained from coffee, and abstainers and low consumers (1-2 cups per day) were therefore included in the lowest (reference) category of intake. For each person belonging to a certain frequency category, observation years at risk of developing breast cancer were computed as the number of years accumulated from the screening examination until withdrawal in the year of diagnosis, at death from a cause other than breast cancer, or at the end of follow-up. Years at risk of developing disease before 51 years were censored when a person reached this age, and years at risk of developing breast cancer at 51

Correspondence: L.J. Vatten, Department of Oncology, University Hospital, N-7006 Trondheim, Norway.
Received 24 October 1989; and in revised form 5 March 1990.

years or later were computed from the time a person reached age 51 and until withdrawal. This procedure allowed comparison of person-time based incidence rates of breast cancer for each category of daily coffee consumption, yielding overall estimates, and distinguishing diagnosis made before and after the age of 51, roughly approximating pre- and postmenopausal breast cancer incidence.

Incidence rate ratios (IRR) were computed as the rate in a specific category of coffee intake divided by the estimated rate in the reference group of lowest intake. The precision of the IRR estimates was assessed by 95% confidence intervals (CI) using Mietinnen's test-based method applying Mantel–Haenszel $\chi^2$ statistics (Kleinbaum et al., 1982). The effect of age was adjusted using the direct method for five-year age categories of person years. Adjustment for other covariables and tests for linear trend followed the Mantel–Haenszel methods (Rothman, 1986). Testing for interaction was performed by fitting the (cumulative incidence) data to multiple logistic models, comparing the maximum likelihood statistics of a model which contained a product between body mass index (two categories) and coffee consumption (four categories) to a model where this term was omitted.

### Results

We first examined the relation of daily coffee consumption with age, body height, body mass index (BMI), total serum cholesterol, and cigarette smoking (Table I), to evaluate whether any one of these factors might distort the association between coffee intake and risk of breast cancer. There was no difference in coffee consumption between age groups, and after adjustment for age there was no relation between coffee intake and body height. We found a weak tendency for more obese women to drink more coffee, and there was a stronger association between total serum cholesterol and coffee consumption, as there was a tendency for women who smoked 10 or more cigarettes per day to report higher intakes of coffee per day than nonsmoking women.

In this cohort, women who were 163 cm (population median) or taller had an increased risk of breast cancer (age-adjusted IRR = 1.5, $P = 0.02$), and relative overweight was inversely related to breast cancer risk. Thus, the age-adjusted IRR of women whose Quetelet's index was 24 (population median) and above was 0.7 ($P = 0.03$) compared to women with Quetelet lower than 24. There was a weak, but not statistically significant negative association between total serum cholesterol and risk of breast cancer. Women whose cholesterol was equal to or above the population median (6.6 mmol $l^{-1}$) had an age-adjusted IRR of 0.8 ($P = 0.20$). There was no difference in risk between women who smoked 10 or more cigarettes per day and nonsmoking women (age-adjusted IRR = 1.1, $P = 0.67$).

We observed an overall weak, but not statistically significant inverse relation between coffee consumption and risk of breast cancer, which was confined to an effect in women who were diagnosed with disease before the age of 51 (Table II). To explore whether this might be an effect of truly premenopausal cancer, only cases diagnosed at age 47 or younger were examined (data not shown), but this did not materially alter the negative association.

Body mass index was inversely related to breast cancer risk, and simultaneously positively associated with coffee consumption. We therefore examined the relation between daily coffee intake and breast cancer risk for lean (Quetelet < 24) and more obese (Quetelet ≥ 24) women separately (Table III). The effect of coffee differed strongly between the two strata of BMI ($\chi^2$ interaction = 10.23, 3 d.f., $P = 0.02$), suggesting that body size might exert an interaction effect on the association between coffee drinking and breast cancer. Among the leaner women, those who drank 5 or more cups per day had an age-adjusted IRR of 0.5 (95% Cl, 0.3 and 0.9), compared to women who had 2 cups or less. The negative relation with coffee consumption yielded a statistically significant test for linear trend ($\chi^2$ trend = 5.07, $P = 0.02$). In the more obese stratum the corresponding age-adjusted IRR was 2.1 (95% Cl, 0.8 and 5.2), but the trend test was not statistically significant for this association ($\chi^2$ trend = 2.90, $P = 0.09$). Further adjustments for cigarette smoking and total serum cholesterol did not materially change these results.

### Discussion

In this prospective study, we detected an association between coffee consumption and breast cancer which was dependent on the body size of the women. In lean women (Quetelet < 24) there was a 50% decreased risk of breast cancer associated with drinking five or more cups of coffee per day compared to drinking two cups or less. In more obese women (Quetelet ≥ 24) the corresponding relation displayed a two-fold increase in breast cancer risk. The test for interaction between coffee intake and body mass was statistically significant ($P = 0.02$) on a multiplicative scale, and the inverse relation between coffee consumption and breast cancer in lean women was precisely estimated and yielded a statistically significant test for linear trend.

Previous studies have displayed contradicting relations between coffee consumption and breast cancer. However, one large case–control study (Lubin et al., 1985) found a negative association, and two prospective studies (Snowdon & Philips, 1984; Jacobsen et al., 1986) also reported inverse effects of coffee, but these were not statistically significant. No study that we are aware of has made a distinction between coffee effects among lean and more obese women.

**Table I** Percentages of women according to categories of daily coffee consumption, within the factors age, body height, body mass index (Quetelet), total serum cholesterol, and current cigarette smoking

| | Daily coffee consumption | | | |
|---|---|---|---|---|
| | *≤ 2 cups* | *3–4 cups* | *5–6 cups* | *≥ 7 cups* |
| Age (at entry) | | | | |
| 35–39 ($n$ = 4,559) | 17 | 37 | 30 | 16 |
| 40–44 ($n$ = 4,506) | 15 | 39 | 31 | 15 |
| 45–51 ($n$ = 5,528) | 15 | 42 | 29 | 13 |
| Body height | | | | |
| < 163 cm | 15 | 41 | 29 | 15 |
| ≥ 163 cm | 16 | 39 | 30 | 15 |
| Body mass index | | | | |
| < 24 kg $m^{-2}$ | 17 | 40 | 29 | 14 |
| ≥ 24 kg $m^{-2}$ | 14 | 40 | 31 | 16 |
| Total serum cholesterol | | | | |
| < 6.6 mmol $l^{-1}$ | 19 | 41 | 28 | 13 |
| ≥ 6.6 mmol $l^{-1}$ | 12 | 39 | 32 | 17 |
| Cigarette smoking | | | | |
| Non-smoker | 21 | 46 | 25 | 8 |
| ≥ 10 cig per day | 9 | 27 | 36 | 29 |

**Table II** Age-adjusted incidence rate ratio (IRR) of breast cancer, according to daily consumption of coffee, for (A) all cases, (B) cases diagnosed before age 51, and (c) cases diagnosed at age 51 or later[a]

| | Daily coffee consumption | | | | |
|---|---|---|---|---|---|
| | *≤ 2 cups* | *3–4 cups* | *5–6 cups* | *≥ 7 cups* | $\chi^2$ |
| A. All cases | | | | | |
| Cases | 27 | 62 | 42 | 21 | |
| Person years | 24611 | 63659 | 48168 | 23759 | |
| Age-adjusted IRR | 1.0 | 0.9 | 0.8 | 0.8 | 0.81 |
| 95% confidence limits | | (0.6, 1.4) | (0.5, 1.3) | (0.5, 1.4) | $P = 0.37$ |
| B. Cases < 51 | | | | | |
| Cases | 18 | 36 | 23 | 14 | |
| Person years | 17089 | 42375 | 32741 | 16665 | |
| Age-adjusted IRR | 1.0 | 0.8 | 0.7 | 0.8 | 0.83 |
| 95% confidence limits | | (0.5, 1.4) | (0.4, 1.2) | (0.4, 1.6) | $P = 0.36$ |
| C. Cases ≥ 51 | | | | | |
| Cases | 9 | 26 | 19 | 7 | |
| Person years | 7773 | 21964 | 15901 | 7380 | |
| Age-adjusted IRR | 1.0 | 1.0 | 1.0 | 0.8 | 0.08 |
| 95% confidence limits | | (0.5, 2.2) | (0.5. 2.3) | (0.3, 2.3) | $P = 0.77$ |

[a]Data are based on 152 cases of breast cancer that developed during a mean follow-up of 14,593 Norwegian women, who were between 35 and 51 years at examination.

**Table III** Age-adjusted incidence rate ratio (IRR) of breast cancer, according to daily coffee consumption in lean women (Quetelet's index < 24), and in more obese (Quetelet's index ≥ 24) women

| | Daily coffee consumption | | | | |
|---|---|---|---|---|---|
| | *< 2 cups* | *3–4 cups* | *5–6 cups* | *≥ 7 cups* | $\chi^2$ |
| Quetelet < 24 | | | | | |
| Cases | 22 | 40 | 17 | 11 | |
| Person years | 13743 | 32355 | 24025 | 11367 | |
| Age-adjusted IRR | 1.0 | 0.8 | 0.4 | 0.6 | 5.07 |
| 95% confidence limits | | (0.5, 1.3) | (0.2, 0.8) | (0.3, 1.2) | $P = 0.02$ |
| Quetelet ≥ 24 | | | | | |
| Cases | 5 | 22 | 25 | 10 | |
| Person years | 10868 | 31304 | 24143 | 12392 | |
| Age-adjusted IRR | 1.0 | 1.5 | 2.3 | 1.8 | 2.33 |
| 95% confidence limits | | (0.6, 4.0) | (0.9, 5.8) | (0.6, 5.4) | $P = 0.13$ |
| | | $\chi^2$ interaction[a] = 10.23, 3 d.f., $P = 0.02$ | | | |

[a]Interaction between coffee consumption (4 categories) and BMI (2 categories).

Approximately 95% of the participating women returned the dietary questionnaire. Although the remaining 5% may represent a selected part of the population with respect to coffee consumption, it seems unlikely that these would materially affect the associations detected with breast cancer. Coffee intake was reliably measured (Solvoll, 1983, 1989) and showed that consumption was generally high, with a population mean of 3.5 cups per day, and that very few women abstained from coffee. Despite a low number of abstainers, there was sufficient variation in consumption to construct 4 separate categories of intake. The 24-hour recall indicated that the accuracy of reporting was fairly high. Although some misclassification could not have been avoided, this would probably be non-differential and not associated with future risk of developing breast cancer. If anything, this suggests that misclassification would produce results that are an underestimate of the real effects of coffee.

A limitation of this study is the lack of information on factors that are known to affect the risk of breast cancer. Apart from being independent risk factors for the disease in the absence of the exposure under study, potentially confounding variables should be associated with the exposure (Rothman, 1986). Consequently, intake of coffee should be associated with variables like age at menarche and age at first full term pregnancy for confounding from these factors to be anticipated in the data. We attempted to make an adjustment for reproductive history by controlling for the effect of occupational status (housewife/not housewife), but this did not materially alter the observed relation with coffee consumption.

There are indications that among premenopausal women those who are lean are at increased risk of developing breast cancer compared to women who are more obese (Willett *et al.*, 1985). In this study the oldest participant was still only 63 years at the end of follow-up. This indicates that a majority of the cases were premenopausal at the onset of disease, and most certainly were premenopausal at initiation and during the preclinical induction phase of disease. Since the protective effect of obesity on the risk of breast cancer appears to be restricted to premenopausal women, this may indicate that there exists a crucial relationship between body mass and ovarian activity. Then the results of this study might suggest that some component contained in coffee (possibly methylxanthines) could have a role to play in this relationship. Whereas large daily doses of coffee may favourably affect the risk of developing breast cancer in lean women, more obese women might achieve a benefit from restricting their daily coffee intake.

This research is based on data made available by the National Health Screening Service and the Cancer Registry of Norway in cooperation with the Division of Dietary Research, the University of Oslo. Dr Vatten is a research fellow of the Norwegian Cancer Society.

**References**

BJARTVEIT, K., FOSS, O.P., GJERVIG, T. & LUND-LARSEN, P.G. (1979). The cardiovascular disease study in Norwegian counties. Background and organization. *Acta Med Scand., Suppl.*, **634**.

BJARTVEIT, K., FOSS, O.P. & GJERVIG, T. (1983). The cardiovascular disease study in Norwegian counties. Results from first screening. *Acta Med. Scand., Suppl.*, **675**.

JACOBSEN, B.K., BJELKE, E., KVÅLE, G. & HEUCH, I. (1986). Coffee drinking mortality, and cancer incidence: results from a Norwegian prospective study. *J. Natl Cancer Inst.*, **76**, 823.

KLEINBAUM, D.G., KUPPER, L.L. & MORGENSTERN, H. (1982). *Epidemiologic Research Principles and Quantitative Methods*. Van Nostrand Reinhold: New York.

LA VECCHIA, C., TALAMANI, R., DECARLI, A., FRANCESCHI, S., PARAZZINI, F. & TOGNONI, F. (1986). Coffee consumption and the risk of breast cancer. *Surgery*, **100**, 477.

LAWSON, D.H., JICK, H. & ROTHMAN, K.J. (1981). Coffee and tea consumption and breast disease. *Surgery*, **90**, 801.

LUBIN, J.H., BURNS, P.E., BLOT, J.W. & 4 others. (1981). Dietary factors and breast cancer risk. *Int. J. Cancer*, **28**, 685.

LUBIN, F., RON, E.M., WAX, Y. & MODAN, B. (1985). Coffee and methylxanthines and breast cancer: a case-control study. *J. Natl Cancer Inst.*, **74**, 569.

MANSEL, R.E., WEBSTER, D.J.T., BURR, M. & ST. LEER, S. (1982). Is there a relationship between coffee consumption and breast disease? *Br. J. Surg.*, **69**, 295.

MINTON, J.P., ABOU-ISSA, H., FOECKING, M.K. & SRIRAM, M.G. (1983). Caffeine and unsaturated fat significantly promotes DMBA-induced breast cancer in rats. *Cancer*, **51**, 1249.

ROHAN, T.E. & MCMICHAEL, A.J. (1988). Methylxanthines and breast cancer. *Int. J. Cancer*, **41**, 390.

ROHAN, T.E. & BAIN, C.J. (1987). Diet in the etiology of breast cancer. *Epidemiol. Rev.*, **9**, 120.

ROSENBERG, L., MILLER, D.R., HELMRICH, S.P. & 4 others (1985). Breast cancer and the consumption of coffee. *Am. J. Epidemiol.*, **122**, 391.

ROTHMAN, K.J. (1986). *Modern Epidemiology*. Little, Brown & Company: Boston.

ROTHWELL, K. (1974). Dose-related inhibition of chemical carcinogenesis in mouse skin by caffeine. *Nature*, **252**, 69.

SNOWDON, D.A. & PHILLIPS, R.L. (1984). Coffee consumption and risk of fatal cancers. *Am. J. Public Health*, **74**, 820.

SOLVOLL, K. (1983). Comparison of dietary data from a self-administered questionnaire and 24 hour recall. Report no. 31 from the Division of Dietary Research, the University of Oslo, Norway.

SOLVOLL, K., SELMER, R., LØKEN, E.B., FOSS, O.P. & TRYGG, K. (1989). Coffee, dietary habits, and serum cholesterol among men and women 35–49 years of age. *Am. J. Epidemiol.*, **129**, 1277.

WILLETT, W.C., BROWNE, M.L., BAIN, C. & 6 others (1985). Relative weight and risk of breast cancer among premenopausal women. *Am. J. Epidemiol.*, **122**, 731.